# Ethyl 2-cyano-2-{(*Z*)-2-[2,2-di­cyano-1-(4-methyl­phen­yl)eth­yl]cyclo­hexyl­idene}acetate

**DOI:** 10.1107/S2414314621005009

**Published:** 2021-05-14

**Authors:** S. Sivapriya, S. Priyanka, M. Gopalakrishnan, H. Manikandan, S. Selvanayagam

**Affiliations:** a Department of Chemistry, Annamalai University, Annamalainagar, Chidambaram 608 002, India; b PG & Research Department of Physics, Government Arts College, Melur 625 106, India; Goethe-Universität Frankfurt, Germany

**Keywords:** crystal structure, cyclo­hexyl­idene derivatives, chair conformation, inter­molecular C—H⋯N hydrogen bonds

## Abstract

In the title compound, C_22_H_23_N_3_O_2_, the cyclo­hexane ring adopts a chair conformation. The methyl­phenyl ring is oriented at an angle of 36.2 (1)° with respect to the best plane of cyclo­hexane moiety. In the crystal, mol­ecules associate *via* C—H⋯N hydrogen bonds, forming a three-dimensional network.

## Abstract 2

In the crystal of the title cyclo­hexyl­idene derivative, mol­ecules associate *via* C—H⋯N hydrogen bonds, forming a three-dimensional network.

## Graphical abstract

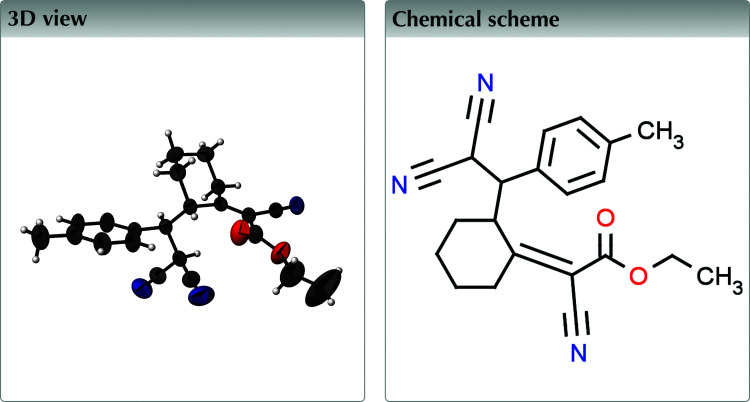

## Structure description

Cyclo­hexyl­idene derivatives possess a wide range of biological activities including anti­bacterial (Gupta & Narayana, 1997 ▸), anti­viral (Ulusoy Guzeldemirci *et al.*, 2016 ▸), anti­tuberculatic and anti-inflammatory (Kabir *et al.*, 2008 ▸). As part of our studies in this area, we have undertaken a single-crystal X-ray diffraction study for the title compound, and the results are presented here.

**Figure 1:**
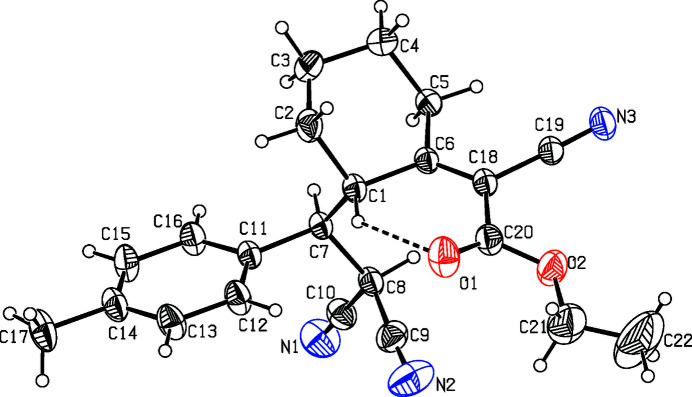
The mol­ecular structure of the title compound, with atom labelling. Displacement ellipsoids are drawn at the 30% probability level. The intramolecular C—H⋯O hydrogen bond is shown as a dashed line.

The methyl­phenyl ring is oriented at an angle of 36.2 (1)° with respect to the best plane of cyclo­hexane moiety. The cyclo­hexane ring adopts a chair conformation, the puckering parameters (Cremer & Pople, 1975 ▸) are: *q*_2_ = 0.001 (1) Å, *q*_3_ = −0.562 (2) Å, *Q*_T_ = 0.562 (2) Å and θ = 179.0 (1)°. Atoms C1 and C4 deviate by 0.681 (1) and −0.652 (1) Å, respectively, from the least-squares plane through the remaining four atoms. An intra­molecular C—H⋯O hydrogen bond is observed (Table 1 ▸), which generates an *S*(6) ring (Fig. 1 ▸).

**Figure 2:**
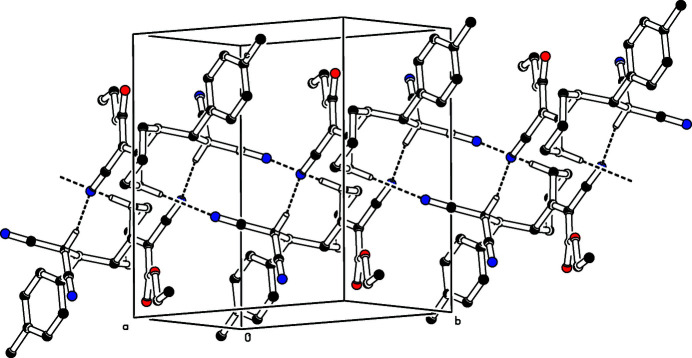
The crystal packing of the title compound viewed down the *a* axis. The C—H⋯N hydrogen bonds (see Table 1 ▸) are shown as dashed lines. For clarity, H atoms not involved in these inter­actions have been omitted.

In the crystal, mol­ecules associate *via* pairwise C5—H5*A*⋯N1^i^ hydrogen bonds into inversion dimers with an 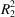 (16) loop motif. In addition, C8—H8⋯N3^ii^ hydrogen bonds form a 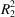 (16) graph-set motif (Fig. 2 ▸).

**Figure 3:**
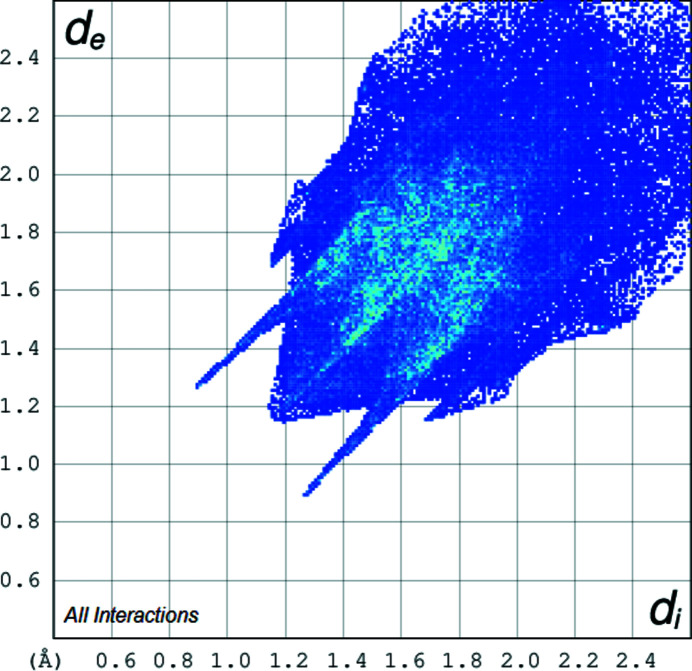
The two-dimensional fingerprint plot for the title compound depicting the overall contribution by the various contacts.

The two-dimensional fingerprint plots (Spackman & Jayatilaka, 2009 ▸) of the mol­ecule, created using *Crystal Explorer 17* (Turner *et al.*, 2017 ▸) for the contacts contributing to the Hirshfeld surface are shown in Figs. 3 ▸–5 ▸ ▸. The analysis reveals that H⋯H contacts (45.7%) and N⋯H/H⋯N contacts (29.8%) are the main contributors to the crystal packing, followed by C⋯H/H⋯C (14%) and O⋯H/H⋯O (7.8%) contacts.

## Synthesis and crystallization

A mixture of 2-amino-4-(*p*-tol­yl)octa­hydro­naphthalene-1,3,3(2*H*)-tricarbo­nitrile (0.01 mol), formic acid (5 mL) and a catalytic amount of concentrated HCl was refluxed for 16 h and the reaction mixture was allowed to cool. The reaction mixture was poured onto crushed ice and the solid that separated was filtered, dried and recrystallized using ethanol and water as mixed solvents.

## Refinement

Crystal data, data collection and structure refinement details are summarized in Table 2 ▸.

## Supplementary Material

Crystal structure: contains datablock(s) I, shelx. DOI: 10.1107/S2414314621005009/bt4111sup1.cif

Structure factors: contains datablock(s) I. DOI: 10.1107/S2414314621005009/bt4111Isup2.hkl

Supporting information file. DOI: 10.1107/S2414314621005009/bt4111Isup3.cml

CCDC reference: 2057162

Additional supporting information:  crystallographic information; 3D view; checkCIF report

## Figures and Tables

**Figure 4:**
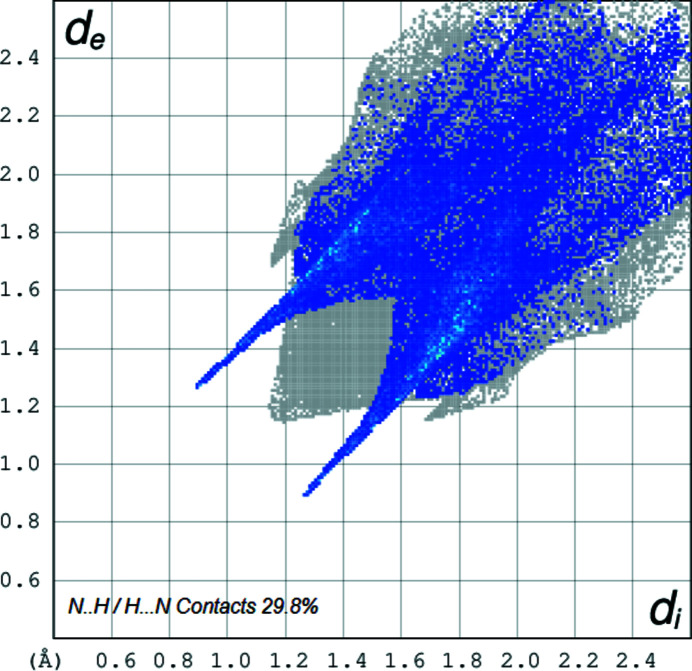
The two-dimensional fingerprint plot for the title compound depicting the contribution of the N⋯H contacts.

**Figure 5:**
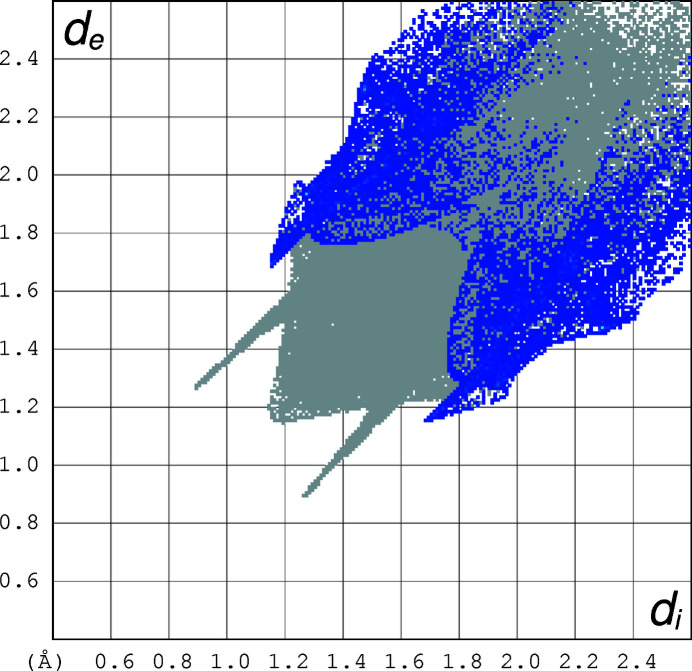
The two-dimensional fingerprint plot for the title compound depicting the contribution of the C⋯H contacts.

**Table 1:** Hydrogen-bond geometry (Å, °)

*D*—H⋯*A*	*D*—H	H⋯*A*	*D*⋯*A*	*D*—H⋯*A*
C1—H1⋯O1	0.98	2.23	3.001 (2)	135
C5—H5*A*⋯N1^i^	0.97	2.57	3.536 (3)	172
C8—H8⋯N3^ii^	0.98	2.27	3.221 (2)	163

Symmetry codes: (i) 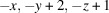 ; (ii) 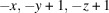 .

**Table 2:** Experimental details

Crystal data	
Chemical formula	C_22_H_23_N_3_O_2_
*M*_r_	361.43
Crystal system, space group	Triclinic, *P* 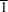
Temperature (K)	296
*a*, *b*, *c* (Å)	8.4465 (3), 10.2960 (4), 12.1699 (4)
α, β, γ (°)	91.240 (1), 95.072 (1), 92.102 (1)
*V* (Å^3^)	1053.17 (7)
*Z*	2
Radiation type	Mo *K*α
μ (mm^−1^)	0.07
Crystal size (mm)	0.24 × 0.21 × 0.19

Data collection	
Diffractometer	Bruker SMART APEX CCD
No. of measured, independent and observed [*I* > 2σ(*I*)] reflections	28133, 6098, 4212
*R*_int_	0.033
(sin θ/λ)_max_ (Å^−1^)	0.704

Refinement	
*R*[*F* ^2^ > 2σ(*F* ^2^)], *wR*(*F* ^2^), *S*	0.064, 0.211, 0.97
No. of reflections	6098
No. of parameters	244
H-atom treatment	H-atom parameters constrained
Δρ_max_, Δρ_min_ (e Å^−3^)	0.38, −0.31

Computer programs: *SMART* and, *SAINT* (Bruker, 2008 ▸), *SHELXS97* (Sheldrick, 2008 ▸), *SHELXL2018/3* (Sheldrick, 2008 ▸), *ORTEP-3 for Windows* (Farrugia, 2012 ▸) and *PLATON* (Spek, 2020 ▸).

## full crystallographic data

### Crystal data

**Table:**

C_22_H_23_N_3_O_2_	*Z* = 2
*M**_r_* = 361.43	*F*(000) = 384
Triclinic, *P*1	*D*_x_ = 1.140 Mg m^−^^3^
*a* = 8.4465 (3) Å	Mo *K*α radiation, λ = 0.71073 Å
*b* = 10.2960 (4) Å	Cell parameters from 18428 reflections
*c* = 12.1699 (4) Å	θ = 2.8–28.2°
α = 91.240 (1)°	µ = 0.07 mm^−^^1^
β = 95.072 (1)°	*T* = 296 K
γ = 92.102 (1)°	Block, colourless
*V* = 1053.17 (7) Å^3^	0.24 × 0.21 × 0.19 mm

### Data collection

**Table:**

Bruker SMART APEX CCD area-detector diffractometer	*R*_int_ = 0.033
Radiation source: fine-focus sealed tube	θ_max_ = 30.0°, θ_min_ = 2.6°
ω and φ scans	*h* = −11→11
28133 measured reflections	*k* = −14→14
6098 independent reflections	*l* = −16→17
4212 reflections with *I* > 2σ(*I*)	

### Refinement

**Table:**

Refinement on *F*^2^	Primary atom site location: structure-invariant direct methods
Least-squares matrix: full	Hydrogen site location: inferred from neighbouring sites
*R*[*F*^2^ > 2σ(*F*^2^)] = 0.064	H-atom parameters constrained
*wR*(*F*^2^) = 0.211	*w* = 1/[σ^2^(*F*_o_^2^) + (0.1037*P*)^2^ + 0.3202*P*] where *P* = (*F*_o_^2^ + 2*F*_c_^2^)/3
*S* = 0.97	(Δ/σ)_max_ < 0.001
6098 reflections	Δρ_max_ = 0.38 e Å^−^^3^
244 parameters	Δρ_min_ = −0.31 e Å^−^^3^
0 restraints	

### Special details

**Table:**

Geometry. All esds (except the esd in the dihedral angle between two l.s. planes) are estimated using the full covariance matrix. The cell esds are taken into account individually in the estimation of esds in distances, angles and torsion angles; correlations between esds in cell parameters are only used when they are defined by crystal symmetry. An approximate (isotropic) treatment of cell esds is used for estimating esds involving l.s. planes.
Refinement. H atoms were placed in idealized positions and allowed to ride on their parent atoms: C—H = 0.93–0.98 Å, with *U*_iso_(H) = 1.5*U*_eq_(C) for methyl H atoms and 1.2*U*_eq_(C) for other H atoms.

### Fractional atomic coordinates and isotropic or equivalent isotropic displacement parameters (Å^2^)

**Table:**

	*x*	*y*	*z*	*U*_iso_*/*U*_eq_	
O1	0.2013 (2)	0.55320 (15)	0.86132 (11)	0.0772 (4)	
O2	0.0185 (2)	0.42057 (16)	0.77154 (13)	0.0819 (5)	
N1	−0.1230 (3)	1.0587 (2)	0.60489 (18)	0.0857 (6)	
N2	−0.0870 (3)	0.7604 (3)	0.8407 (2)	0.1067 (8)	
N3	0.11934 (19)	0.34667 (15)	0.52121 (14)	0.0616 (4)	
C1	0.30484 (18)	0.75924 (14)	0.70937 (13)	0.0443 (3)	
H1	0.285441	0.734845	0.784544	0.053*	
C2	0.4861 (2)	0.78324 (19)	0.70392 (18)	0.0628 (5)	
H2A	0.521171	0.859385	0.749172	0.075*	
H2B	0.541326	0.709455	0.734562	0.075*	
C3	0.5315 (2)	0.8033 (2)	0.5874 (2)	0.0732 (6)	
H3A	0.486124	0.882452	0.559229	0.088*	
H3B	0.646316	0.813127	0.588507	0.088*	
C4	0.4726 (2)	0.6896 (2)	0.5118 (2)	0.0700 (5)	
H4A	0.528715	0.612669	0.534317	0.084*	
H4B	0.495422	0.708172	0.436902	0.084*	
C5	0.2929 (2)	0.66303 (15)	0.51486 (13)	0.0489 (4)	
H5A	0.235573	0.735033	0.482858	0.059*	
H5B	0.260103	0.585006	0.471424	0.059*	
C6	0.25320 (16)	0.64621 (13)	0.63136 (12)	0.0411 (3)	
C7	0.21454 (17)	0.88396 (13)	0.68062 (12)	0.0417 (3)	
H7	0.244434	0.910752	0.608248	0.050*	
C8	0.03147 (19)	0.85589 (15)	0.66844 (14)	0.0488 (4)	
H8	0.009240	0.789898	0.609301	0.059*	
C9	−0.0339 (2)	0.8042 (2)	0.76680 (19)	0.0646 (5)	
C10	−0.0551 (2)	0.97122 (18)	0.63408 (15)	0.0576 (4)	
C11	0.26493 (18)	0.99501 (14)	0.76145 (12)	0.0440 (3)	
C12	0.2656 (3)	0.98255 (17)	0.87475 (14)	0.0602 (5)	
H12	0.231616	0.904205	0.903138	0.072*	
C13	0.3163 (3)	1.08555 (19)	0.94631 (15)	0.0662 (5)	
H13	0.315364	1.074856	1.021940	0.079*	
C14	0.3677 (2)	1.20274 (18)	0.90808 (16)	0.0602 (5)	
C15	0.3642 (3)	1.21591 (18)	0.79527 (17)	0.0660 (5)	
H15	0.397002	1.294798	0.767239	0.079*	
C16	0.3129 (2)	1.11375 (16)	0.72261 (15)	0.0563 (4)	
H16	0.310842	1.125603	0.646965	0.068*	
C17	0.4252 (3)	1.3131 (2)	0.9871 (2)	0.0852 (7)	
H17A	0.456292	1.387009	0.946141	0.128*	
H17B	0.341186	1.335986	1.031229	0.128*	
H17C	0.514778	1.286442	1.034240	0.128*	
C18	0.18375 (18)	0.53484 (14)	0.66384 (12)	0.0437 (3)	
C19	0.14525 (18)	0.43040 (14)	0.58413 (13)	0.0461 (3)	
C20	0.1382 (2)	0.50714 (16)	0.77718 (15)	0.0548 (4)	
C21	−0.0385 (4)	0.3775 (3)	0.8773 (3)	0.1121 (11)	
H21A	0.051164	0.353762	0.927149	0.135*	
H21B	−0.090609	0.448219	0.911649	0.135*	
C22	−0.1466 (7)	0.2695 (6)	0.8578 (4)	0.230 (4)	
H22A	−0.182981	0.242201	0.926523	0.345*	
H22B	−0.235819	0.293629	0.809141	0.345*	
H22C	−0.094368	0.199386	0.824606	0.345*	

### Atomic displacement parameters (Å^2^)

**Table:**

	*U*^11^	*U*^22^	*U*^33^	*U*^12^	*U*^13^	*U*^23^
O1	0.1095 (12)	0.0724 (9)	0.0462 (7)	−0.0169 (8)	−0.0038 (7)	0.0028 (6)
O2	0.0936 (11)	0.0866 (10)	0.0649 (9)	−0.0352 (9)	0.0220 (8)	−0.0082 (7)
N1	0.0845 (13)	0.0805 (12)	0.0916 (14)	0.0291 (10)	−0.0066 (10)	0.0086 (10)
N2	0.0828 (14)	0.1213 (19)	0.122 (2)	0.0053 (13)	0.0354 (14)	0.0376 (16)
N3	0.0638 (9)	0.0504 (8)	0.0681 (10)	−0.0032 (6)	−0.0017 (7)	−0.0148 (7)
C1	0.0461 (7)	0.0394 (7)	0.0454 (7)	−0.0001 (5)	−0.0041 (6)	−0.0053 (6)
C2	0.0452 (8)	0.0555 (9)	0.0841 (13)	0.0001 (7)	−0.0095 (8)	−0.0148 (9)
C3	0.0482 (9)	0.0661 (11)	0.1056 (17)	−0.0106 (8)	0.0168 (10)	−0.0112 (11)
C4	0.0612 (11)	0.0656 (11)	0.0858 (14)	−0.0039 (9)	0.0272 (10)	−0.0125 (10)
C5	0.0544 (9)	0.0440 (7)	0.0484 (8)	−0.0004 (6)	0.0087 (6)	−0.0049 (6)
C6	0.0386 (7)	0.0381 (6)	0.0454 (7)	0.0034 (5)	−0.0021 (5)	−0.0044 (5)
C7	0.0458 (7)	0.0383 (7)	0.0396 (7)	−0.0001 (5)	−0.0014 (5)	−0.0057 (5)
C8	0.0475 (8)	0.0433 (7)	0.0537 (9)	0.0030 (6)	−0.0040 (6)	−0.0112 (6)
C9	0.0506 (9)	0.0612 (10)	0.0824 (13)	0.0027 (8)	0.0066 (9)	0.0061 (10)
C10	0.0557 (9)	0.0585 (10)	0.0568 (10)	0.0076 (8)	−0.0053 (7)	−0.0078 (8)
C11	0.0475 (8)	0.0391 (7)	0.0439 (7)	0.0001 (6)	−0.0020 (6)	−0.0071 (6)
C12	0.0866 (13)	0.0476 (8)	0.0446 (8)	−0.0081 (8)	0.0010 (8)	−0.0054 (7)
C13	0.0893 (14)	0.0608 (10)	0.0457 (9)	−0.0025 (9)	−0.0044 (9)	−0.0133 (8)
C14	0.0635 (10)	0.0509 (9)	0.0621 (10)	0.0011 (7)	−0.0111 (8)	−0.0184 (8)
C15	0.0853 (13)	0.0431 (8)	0.0664 (11)	−0.0135 (8)	−0.0021 (10)	−0.0064 (8)
C16	0.0739 (11)	0.0453 (8)	0.0480 (8)	−0.0082 (7)	0.0008 (8)	−0.0031 (7)
C17	0.1015 (17)	0.0665 (12)	0.0806 (15)	−0.0079 (12)	−0.0192 (12)	−0.0301 (11)
C18	0.0449 (7)	0.0394 (7)	0.0454 (7)	0.0007 (5)	−0.0015 (6)	−0.0044 (6)
C19	0.0447 (7)	0.0409 (7)	0.0515 (8)	0.0009 (6)	−0.0005 (6)	−0.0028 (6)
C20	0.0654 (10)	0.0459 (8)	0.0528 (9)	−0.0025 (7)	0.0055 (8)	−0.0002 (7)
C21	0.133 (3)	0.122 (2)	0.0823 (18)	−0.037 (2)	0.0367 (17)	0.0020 (16)
C22	0.243 (6)	0.294 (8)	0.152 (4)	−0.161 (6)	0.090 (4)	−0.004 (4)

### Geometric parameters (Å, º)

**Table:**

O1—C20	1.192 (2)	C8—C10	1.466 (2)
O2—C20	1.320 (2)	C8—C9	1.465 (3)
O2—C21	1.484 (3)	C8—H8	0.9800
N1—C10	1.132 (2)	C11—C16	1.380 (2)
N2—C9	1.135 (3)	C11—C12	1.387 (2)
N3—C19	1.143 (2)	C12—C13	1.388 (2)
C1—C6	1.5125 (19)	C12—H12	0.9300
C1—C7	1.548 (2)	C13—C14	1.372 (3)
C1—C2	1.550 (2)	C13—H13	0.9300
C1—H1	0.9800	C14—C15	1.380 (3)
C2—C3	1.518 (3)	C14—C17	1.511 (2)
C2—H2A	0.9700	C15—C16	1.391 (2)
C2—H2B	0.9700	C15—H15	0.9300
C3—C4	1.514 (3)	C16—H16	0.9300
C3—H3A	0.9700	C17—H17A	0.9600
C3—H3B	0.9700	C17—H17B	0.9600
C4—C5	1.537 (3)	C17—H17C	0.9600
C4—H4A	0.9700	C18—C19	1.441 (2)
C4—H4B	0.9700	C18—C20	1.495 (2)
C5—C6	1.498 (2)	C21—C22	1.415 (5)
C5—H5A	0.9700	C21—H21A	0.9700
C5—H5B	0.9700	C21—H21B	0.9700
C6—C18	1.351 (2)	C22—H22A	0.9600
C7—C11	1.5158 (18)	C22—H22B	0.9600
C7—C8	1.556 (2)	C22—H22C	0.9600
C7—H7	0.9800		
			
C20—O2—C21	117.29 (18)	N2—C9—C8	177.5 (3)
C6—C1—C7	112.65 (11)	N1—C10—C8	178.2 (2)
C6—C1—C2	107.22 (13)	C16—C11—C12	117.76 (14)
C7—C1—C2	110.65 (13)	C16—C11—C7	119.73 (14)
C6—C1—H1	108.7	C12—C11—C7	122.51 (14)
C7—C1—H1	108.7	C11—C12—C13	120.84 (17)
C2—C1—H1	108.7	C11—C12—H12	119.6
C3—C2—C1	113.00 (15)	C13—C12—H12	119.6
C3—C2—H2A	109.0	C14—C13—C12	121.55 (18)
C1—C2—H2A	109.0	C14—C13—H13	119.2
C3—C2—H2B	109.0	C12—C13—H13	119.2
C1—C2—H2B	109.0	C13—C14—C15	117.60 (15)
H2A—C2—H2B	107.8	C13—C14—C17	120.90 (19)
C4—C3—C2	111.15 (17)	C15—C14—C17	121.50 (19)
C4—C3—H3A	109.4	C14—C15—C16	121.44 (17)
C2—C3—H3A	109.4	C14—C15—H15	119.3
C4—C3—H3B	109.4	C16—C15—H15	119.3
C2—C3—H3B	109.4	C11—C16—C15	120.78 (16)
H3A—C3—H3B	108.0	C11—C16—H16	119.6
C3—C4—C5	111.29 (15)	C15—C16—H16	119.6
C3—C4—H4A	109.4	C14—C17—H17A	109.5
C5—C4—H4A	109.4	C14—C17—H17B	109.5
C3—C4—H4B	109.4	H17A—C17—H17B	109.5
C5—C4—H4B	109.4	C14—C17—H17C	109.5
H4A—C4—H4B	108.0	H17A—C17—H17C	109.5
C6—C5—C4	110.22 (15)	H17B—C17—H17C	109.5
C6—C5—H5A	109.6	C6—C18—C19	119.19 (14)
C4—C5—H5A	109.6	C6—C18—C20	126.34 (13)
C6—C5—H5B	109.6	C19—C18—C20	114.46 (13)
C4—C5—H5B	109.6	N3—C19—C18	177.98 (17)
H5A—C5—H5B	108.1	O1—C20—O2	124.07 (18)
C18—C6—C5	121.57 (13)	O1—C20—C18	125.98 (17)
C18—C6—C1	123.37 (14)	O2—C20—C18	109.94 (15)
C5—C6—C1	114.98 (12)	C22—C21—O2	109.9 (3)
C11—C7—C1	111.82 (11)	C22—C21—H21A	109.7
C11—C7—C8	112.83 (12)	O2—C21—H21A	109.7
C1—C7—C8	111.14 (12)	C22—C21—H21B	109.7
C11—C7—H7	106.9	O2—C21—H21B	109.7
C1—C7—H7	106.9	H21A—C21—H21B	108.2
C8—C7—H7	106.9	C21—C22—H22A	109.5
C10—C8—C9	108.96 (15)	C21—C22—H22B	109.5
C10—C8—C7	111.69 (14)	H22A—C22—H22B	109.5
C9—C8—C7	114.82 (14)	C21—C22—H22C	109.5
C10—C8—H8	107.0	H22A—C22—H22C	109.5
C9—C8—H8	107.0	H22B—C22—H22C	109.5
C7—C8—H8	107.0		
			
C6—C1—C2—C3	54.57 (19)	C8—C7—C11—C12	74.0 (2)
C7—C1—C2—C3	−68.63 (18)	C16—C11—C12—C13	−1.4 (3)
C1—C2—C3—C4	−56.0 (2)	C7—C11—C12—C13	178.39 (17)
C2—C3—C4—C5	54.3 (2)	C11—C12—C13—C14	−0.1 (3)
C3—C4—C5—C6	−53.9 (2)	C12—C13—C14—C15	1.3 (3)
C4—C5—C6—C18	−120.06 (16)	C12—C13—C14—C17	−178.9 (2)
C4—C5—C6—C1	56.71 (17)	C13—C14—C15—C16	−0.9 (3)
C7—C1—C6—C18	−117.15 (16)	C17—C14—C15—C16	179.3 (2)
C2—C1—C6—C18	120.89 (16)	C12—C11—C16—C15	1.8 (3)
C7—C1—C6—C5	66.15 (17)	C7—C11—C16—C15	−178.03 (16)
C2—C1—C6—C5	−55.82 (17)	C14—C15—C16—C11	−0.6 (3)
C6—C1—C7—C11	178.52 (12)	C5—C6—C18—C19	−0.8 (2)
C2—C1—C7—C11	−61.47 (17)	C1—C6—C18—C19	−177.25 (13)
C6—C1—C7—C8	51.44 (17)	C5—C6—C18—C20	−179.74 (15)
C2—C1—C7—C8	171.45 (13)	C1—C6—C18—C20	3.8 (2)
C11—C7—C8—C10	57.22 (18)	C21—O2—C20—O1	−0.9 (3)
C1—C7—C8—C10	−176.25 (13)	C21—O2—C20—C18	177.7 (2)
C11—C7—C8—C9	−67.48 (17)	C6—C18—C20—O1	−28.4 (3)
C1—C7—C8—C9	59.05 (17)	C19—C18—C20—O1	152.57 (19)
C1—C7—C11—C16	127.58 (17)	C6—C18—C20—O2	153.07 (17)
C8—C7—C11—C16	−106.25 (18)	C19—C18—C20—O2	−26.0 (2)
C1—C7—C11—C12	−52.2 (2)	C20—O2—C21—C22	−169.7 (4)

### Hydrogen-bond geometry (Å, º)

**Table:**

*D*—H···*A*	*D*—H	H···*A*	*D*···*A*	*D*—H···*A*
C1—H1···O1	0.98	2.23	3.001 (2)	135
C5—H5*A*···N1^i^	0.97	2.57	3.536 (3)	172
C8—H8···N3^ii^	0.98	2.27	3.221 (2)	163

Symmetry codes: (i) −*x*, −*y*+2, −*z*+1; (ii) −*x*, −*y*+1, −*z*+1.
